# The cultural transmission of cooperative norms

**DOI:** 10.3389/fpsyg.2015.01554

**Published:** 2015-10-13

**Authors:** Xinyue Zhou, Yan Liu, Benjamin Ho

**Affiliations:** ^1^Department of Psychology, Sun Yat-Sen University, GuangzhouChina; ^2^Lingnan (University) College, Sun Yat-Sen University, GuangzhouChina; ^3^Department of Economics, Vassar College, Poughkeepsie, NYUSA

**Keywords:** trust, moral licensing, primacy, recency, corporate culture, social norms

## Abstract

Cooperative behavior depends on cultural environment, so what happens when people move from to a new culture governed by a new norm? The dynamics of culture-induced cooperation has not been well understood. We expose lab participants to a sequence of different subject pools while playing a constrained Trust Game. We find prior exposure to different subject pools does in fact influence cooperative behavior; first impressions matter—the primacy effect plays a stronger role than the recency effect; and selfish first impressions matter more than cooperative first impressions—observing selfish behavior by others had a longer-lasting and greater influence on behaviors than observing cooperative behavior by others. Moreover, three consecutive exposures to cooperative environments were needed to neutralize one exposure to a selfish environment.

## Introduction

Cultural norms shape behaviors, but cultural norms are not static. An individual may move from a selfish cultural environment to a cooperative cultural environment and vice versa. Moving to live in another culture has become increasingly common for much of the world’s population, even becoming a way of life for some (Oishi, 2014). Today many people live in a culture drastically different from the one they grew up in. Even within a single culture, people operate under different norms in different spheres of their life: home, school, work, etc. They juggle between multiple social roles. Sometimes people change the place where they work and have to adapt their behaviors based on their observations of the new environment. Moreover, cultural norms can change within firms or organizations or in society as a whole. Groups may shift from being cooperative to being selfish when competition and stresses are high, or they may change from selfish to cooperative. When we come to a new environment or encounter a new situation, how will the norms encountered in previous situations continue to affect our choices?

Descriptive cultural norms are the observations people make of what is commonly done in a particular culture (e.g., most people don’t litter). In this research, we focus on the dynamic impact of changing descriptive norms by providing participants an opportunity to observe and participate in settings with different norms of behavior. Our mechanism seeks to understand the impact of conformity and social influence (Becker, 1991; Bernheim, 1994; for a review, see Moscovici, 1985) as subjects change environments.

Behavior is not only a product of currently activated cultural norms (Bicchieri, 2006). Instead it can also be affected by previously encountered cultural norms. A substantial literature has tried to ascertain the impact of exposures to different societal norms by looking at immigrants’ behavior, and how and when immigrants conform to the normative behaviors of their country of origin or to the normative behavior of their new home. For example, Borjas (2000) shows that immigrants’ economic achievement can be predicted by which country they originally came from. Social norms regarding misconduct can also be carried over from the home culture to the host culture. In a lab setting such as ours, Cox et al. (1991) found that behavior of immigrant minority groups in a prisoners’ dilemma game adhered to domestic norms in neutral conditions, but behaved more like their home culture when cued to think about their minority identity.

Selfish norms seem to be longer lasting and more impactful than cooperative norms. Research has uncovered possible asymmetry between the persistence of selfish cultural norms as environments change compared to the persistence of cooperative social norms. In a study investigating the influence of home culture norms on diplomatic parking tickets issued to UN employees in New York City (Fisman and Miguel, 2007), diplomats stationed in New York who come from a country with high corruption levels received more unpaid parking tickets than diplomats from countries with low corruption levels. Importantly, socialization to the local norm would predict a decline in the rate of parking violations over time for those from high-corruption countries and an increase in parking violations for those from low-corruption countries. Results show the violations frequency did increase with tenure in New York City for diplomats from low-corruption countries. However, diplomats from high-corruption countries did not show declining parking violations over time. While part of their findings can be explained by the enforcement environment, substantial variation in the behavior of diplomats can likely still be attributed to norms. While prior evidence of an asymmetry about the lasting impact of selfish versus cooperative norms is limited, the Fisman and Miguel (2007) provides suggestive evidence that negative social norms may be more powerful and stickier than positive social norms.

The motivating literature for our experimental design lends itself to two sets of questions. The first set asks which set of cultural norms does an individual adhere to when confronted with a sequence of norms from multiple environments. The second asks whether cooperative norms or selfish norms are more influential.

To compare with findings reviewed in the previous section, the primacy effect represents the impact of one’s home environment, whereas the recency effect represents the impact of one’s current environment. Which effect dominates depends on the strength of each stimulus, but in our setting, we expect that the novelty of the first environmental exposure will make primacy stronger than the most recent environmental exposure. We expect this for two reasons. (1) We respond proportional to the novelty of the stimulus. By the time the subject is exposed to the last treatment, the treatment may have become boring and thus the subject may pay less attention to it. (2) Participants in lab experiments tend exhibit a lot of inertia in their behavior. Once they have established a pattern after the first treatment, it becomes more difficult for latter stimuli to change that pattern.

The second set of hypotheses compares the difference in the strength of the primacy effect with the counteracting influence of recent exposures. These hypotheses were inspired by the Fisman and Miguel (2007) finding that people are more likely to assimilate to more selfish norms, than more cooperative norms. The asymmetric persistence between cooperative versus selfish norms can be seen through the lens of moral licensing (Sachdeva et al., 2009; Klotz and Bolino, 2012). In a public goods game where participants learned that others were either more moral or less moral than themselves, Ho et al. (2015) found that people were more likely to respond to information that licensed their own selfish behavior, rather than information that demanded cooperation. Thus, we hypothesize that when exposed to both a cooperative environment and a selfish environment, the selfish effects are more likely to persist.

In this study, we investigate one facet of cultural change: the impact of moving from a selfish environment to a cooperative environment compared to the impact of moving from a cooperative environment to a selfish environment. Cultural norms are difficult to manipulate precisely in the lab (Rousseau, 1990; Marcoulides and Heck, 1993; Schein, 1996). As a result, researchers have relied on ethnographic observations or questionnaire surveys to study cultural norms in organizations (e.g., Schall, 1983; Hofstede et al., 1990; Rousseau, 1990; Schein, 1990; O’Reilly et al., 1991; Chatterjee et al., 1992). Although such observational studies are informative and inspiring, their methods are not well suited for causal inference.

However, there is a more recent tradition of studying culture in a lab context by exposing participants to the play style of others while playing economic games. For example, Falk et al. (2003) and Bardsley and Sausgruber (2005) isolate the conformity effect from reciprocity by exposing participants to information about different groups in order to argue that conformity was driven by social norms, rather than economic incentives. More recently, a number of studies have used similar methods to develop the Social Heuristics Hypothesis (Rand et al., 2012, 2014; Peysakhovich and Rand, 2015). They varied subjects’ experiences in lab settings while playing economic games, to argue that people develop flexible social heuristics that change as they learn through experience. While culture is certainly much more complicated that simple social heuristics and social norm conformity, these lab methods have allowed for controlled experimentation on these important components of culture.

Therefore, we create an experimental setting to manipulate participant’s perceived cultural norms—as motivated by Cialdini et al.’s (2006) definition of descriptive norms—to allow us to examine the dynamics of this one dimension of culture in a controlled and novel way. We give up realism in order to precisely manipulate the variables of interest.

In our design the same participant observes and plays as the responder in a constrained trust game across different cultural environments. Some participants encountered a cooperative environment first and then a selfish environment. Others encountered a selfish environment and then a cooperative one. To achieve this, participants played a constrained trust games with students from several different universities. In order for participants to learn about the cultural norms governing each environment, participants were allowed to observe interactions by others in each new environment they were placed into before participants play the game in that environment themselves.

One concern of manipulating perception of norms in the lab is that we would ideally like to disentangle the effects of norms from the effect of training. It is a well-established finding that cooperation in experimental games tends to decline with repeated play, often attributed to learning (Isaac et al., 1994). Recent work has shown that the tendency toward selfish behavior can be counteracted by the establishment of norms (Reuben and Riedl, 2013). Our experimental design only compares behavior of participants with the same length of exposure to the game, which should hold training effects constant. An additional concern in our design is that the changing environments could be construed as feedback for past behavior. Differences in feedback may affect learning. However, our design was careful to emphasize that the information provided applies only to the new environment they will be playing next, and was not related to the set of choices they just made. Manipulation checks showed that subjects did indeed use the normative information we provided to make inferences about the new school they matched with next.

Another concern for manipulating perception of norms is that individuals differ in the standards they apply in judging others’ behavior, so experimental manipulation needs to be tailored to each individual’s moral standards. For example, a highly cooperative individual may consider an act as selfish, whereas a highly selfish individual may consider the same act as cooperative. In order to control for the variation in behavior standards and maximize the power of our experimental design, the interactions students were shown were selected based on their own past behavior. Blanco et al. (2010) argue that a subject defines as “kindness” any observed behavior where others act more pro-socially than the subject would have acted herself. Using this idea, we created a “cooperative” environment, by selectively showing interactions where 80% of the others responded more pro-socially that the subject had in a pre-test. We created a “selfish” environment by showing interactions where 80% of the others responded more selfishly. This allows the experimental manipulation to be tailored to each individual’s moral standards and was done in a way that exposure to a cooperative environment and a selfish environment was perfectly symmetrical for each subject.

Two studies were conducted to examine the effect of dynamic cultural norms, with Study 1 examining whether norm changes influence the level of cooperativeness and Study 2 examining how long normative influences persist.

## Study 1: Primacy versus Recency

### Method

#### Participants

One-hundred students (65% female, 35% male) from Sun Yat-sen University participated in the study for a ¥10 payment plus 1% of the payment earned from the economic game they played. Their ages ranged from 18 to 25 years (*M* = 20.26, *SD* = 1.55). This sample size was determined based on pilot work that indicated the magnitude of the expected effect. Participants were randomly assigned to one of four groups that were exposed to different cultural environments in different orders: CS (cooperative then selfish; *n* = 25); SC (selfish then cooperative; *n* = 25), CC (cooperative in both rounds; *n* = 25), and SS (selfish in both rounds; *n* = 25). This study was approved by the Sun Yat-Sen University Human Research Ethics Committee. All participants were fully informed of the nature of the experiment and signed an informed consent to participate and gave permission to use their data. Participants were also recruited in partnership with four other university labs to serve as Investors for our participants who each gave consent for their data to be used for experiments, however, our analysis is exclusively on the behavior of the Receivers.

#### Procedure

Participants were tested individually. Upon entering the laboratory, each participant was escorted to a private subject room. After completing consent forms, they were given instructions which ensured them their personal identity would remain anonymous. Participants were told they would interact with other students from other universities when playing the game.

After reading and signing consent forms, participants were given instructions on the game they were about to play. They were told there would be two roles in the game: Investor and Receiver. Each round of the game involves two players, an Investor and a Receiver. Participants were told they were assigned to play as a Receiver.

Participants then read a description of the game. The Investor was given ¥10 and then the Investor would give the ¥10 to the Receiver. Our game description is a little different from the trust game developed by Berg et al. (1995) in that the investor is not given a choice to invest or not. The game is framed in a way that participants assume that all the investors would give ¥10 to the Receiver. So the game is akin to a Dictator Game (Kahneman et al., 1986) where the Receiver acts as the dictator, but framed as a Trust Game (Berg et al., 1995). This framing was used because we are particularly interested in the cultural norms of reciprocity.

When the investor gives the ¥10 to the Receiver, the ¥10 is tripled. Thus, the participant receives ¥30 in total. The participant then must decide how much of the ¥30, if any, to return to the Investor. The instructions state the participant could return any amount, from ¥0 to ¥30. Any amount not returned became the basis of the participant’s payoff for that round in the game.

The instructions informed participants they would make decisions in many rounds but they would always play in a role of Receiver. We emphasized that in each round, they would be playing with different investors, and that these investors were from different universities. Participants would not be given the exact names of these universities. Following the instructions, participants completed a “quiz” designed to ensure they understood key aspects of the procedures. A research assistant was available to address any misunderstandings or confusion.

##### Experimental conditions

**FIGURE 1:**
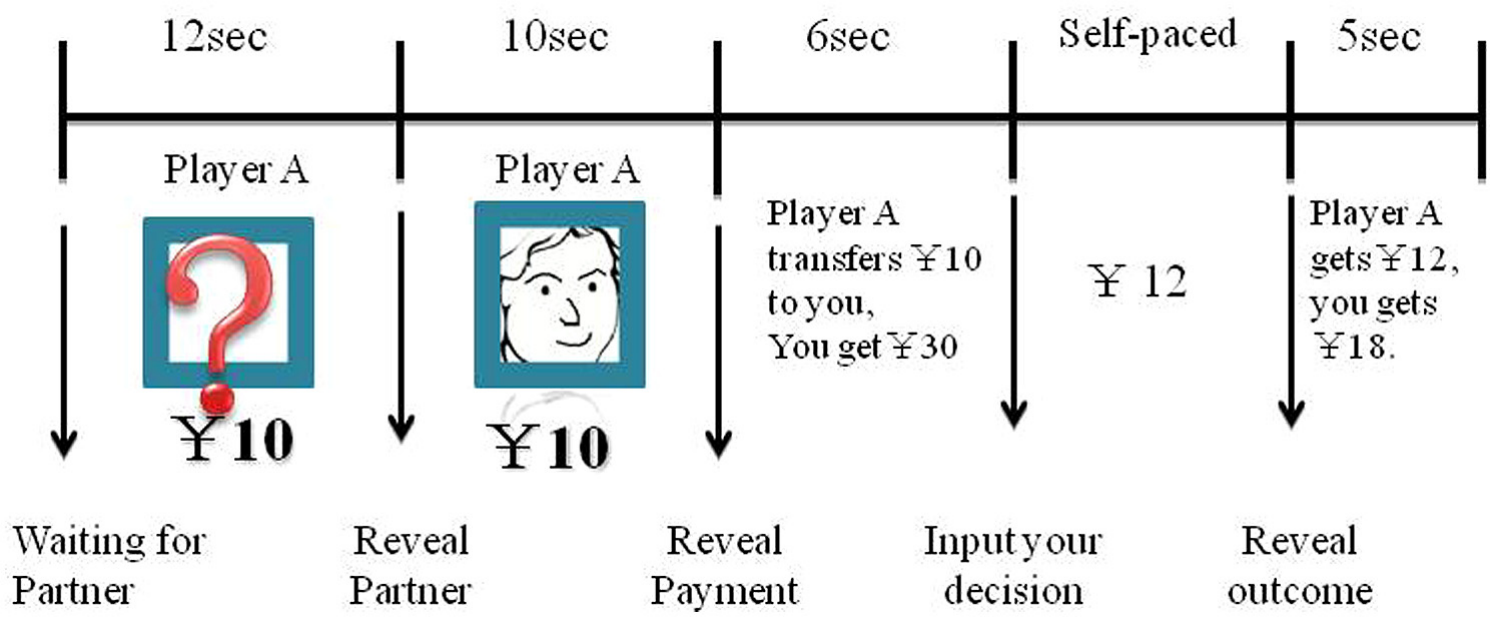
**Example of a single round for the Trust Game.** Each round began with a 12s waiting for partner. The participant then saw the head portrait (created by the authors using software Corel painter 12, Corel software, 2011) of their partner for 10s. Next, participants saw the amount of payment returned by the partner for 6s, after which they decided how much money they would transfer to the partner.

**FIGURE 2:**
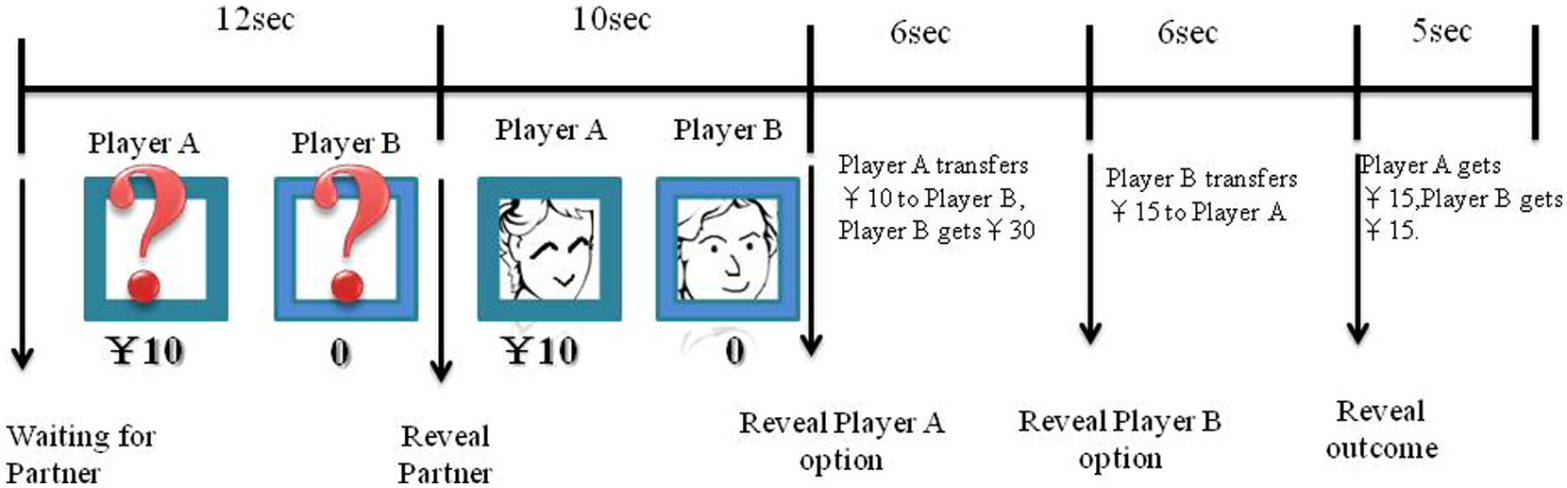
**Example of a single round for the video.** Each round lasted 39s. Each round began with a 12s waiting for partner. The participant then viewed the head portrait of two players (Player A and Player B) for 10s. Next, participants viewed the amount of payment returned by the Player A for 6s, after which Player B decided how much money they would transfer to Player A for a further 6s.

Participants played the game with students from four different universities. They played ten rounds of the game with students from each university and each round was played with a different investor. That is, students played with students from University 1 for 10 rounds, those from University 2 for 10 rounds, those from University 3 for 10 rounds, and finally those from University 4 for 10 rounds (see **Figure 1**, for example). For University 2 and University 3, we had extra information to show participants before they played. We showed participants the play records of other students from University 2 and University 3 playing the game amongst themselves, so that our participants could view the amount of payment returned when two other students were playing the game (see **Figure 2**, for example). Before playing with students from University 2, participants viewed ten rounds of previous game play among students from University 2 (i.e., both Investor and Receiver were from University 2). The same was true before they played with University 3. Participants were told specifically that the students they played the game with were different from the ones who were playing in the recorded interaction, but were from the same university.

These recordings were actual recordings selected from a data pool created by having other university students play the game amongst themselves. They were selected to show students either a more cooperative environment or a more selfish environment than the student’s baseline behavior.

For participants in the C-S condition, they would find Receivers from University 2 typically behaved fairly and Receivers from University 3 typically behaved selfishly in the video that participants viewed. For those in the S-C condition, University 2 was selfish and University 3 was cooperative. For those in the S-S and C-C conditions, University 2 and 3 were both selfish or both cooperative.

A typical session proceeded as follows. Each session lasted approximately 1 h. First, participants played ten rounds as the responder with different investors from University 1 (without learning any information about University 1 before play). Their decisions from this round were used as the basis for constructing a cooperative environment or a selfish environment for them to view later. If they were assigned to a condition that involved ten rounds in a cooperative environment, we randomly selected 8 interaction decisions from the data pool that were each 1 yuan more than participant’s own decisions. For the remaining two trials, we selected interaction decisions from the data pool that were each 1 yuan less than participants’ own decisions to show. On the other hand, if they were to view ten rounds in a selfish environment, we randomly selected eight interaction decisions from the data pool that were 1 yuan less. For the remaining two, we used interactions that were 1 yuan more. The presentation order in the slides view was randomized so no participants suspected the manipulation. This manipulation was designed to maximize the power of the treatment conditions by ensuring a selfish environment was always more selfish than participants’ initial tendencies and a cooperative environment was always more cooperative than participants’ initial tendency.

As a manipulation check, we recruited 40 additional undergraduate students and assigned them to the same four treatments. After they were exposed to each ten round recording we asked “When you viewed ten rounds from previous game playing among students from University 2, did you think the Receivers from University 2 behaved fairly? Responses were made using a scale from 1 (very selfish) to 7 (very cooperative).”

Participants in the cooperative condition reported perceiving the people they played with to be more cooperative (*M* = 5.33, *SD* = 1.42) than did participants in the selfish condition [*M* = 2.58, *SD* = 1.36), *t*(78) = 8.85, *p* < 0.001.

##### Payment and debriefing

Following previous work (Bottom, 1998), the instructions explained that participants will receive a ¥10 base payment plus 1% of the money they earned from the experimental games so it was important they consider each decision very carefully. At the end of the study, participants were paid an average of ¥18 (the range was from ¥14 to ¥22). Afterward, the experimenter explained the study in detail and assessed each participant’s suspicion using a funnel briefing procedure.

##### Dependent measure

Our dependent measures were four indexes of cooperativeness, measured by the average amount per round returned to students from each of the four universities across 10 rounds. Participants played games with those from University 1 first and University 4 last. Participants did not observe any information about University 1 and University 4 before they played with students from those schools. Preferences for cooperation with students from University 4 should not be directly affected by observations for University 2 or 3. In contrast, participants observed recordings from University 2 and University 3 so their cooperativeness with students from these two schools might be influenced by desires to conform to the behavior norms in those schools. We use the average amount they returned to investors from University 1 as the baseline cooperativeness measure and the average amount returned to University 4, the final cooperativeness measure, as the dependent variable of interest.

### Results

**FIGURE 3:**
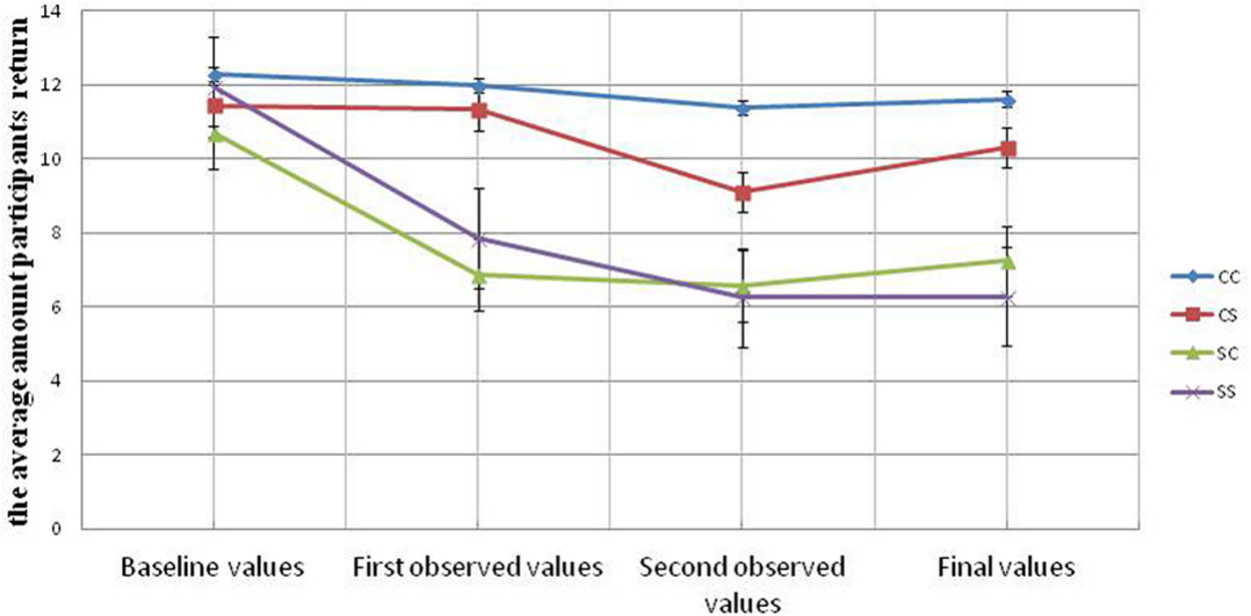
**Average return amounts of participants from each of the four universities across 10 rounds in Study 1.** Error bars represent the standard error of the mean.

**Figure 3** and **Table 1** reported four cooperativeness indices (i.e., average return amounts) for each of the four universities the students played with. As can be seen from **Figure 3**, the initial cooperativeness level was not significantly different among groups, *F*(3,96) = 0.66, *p* > 0.250.

**Table 1:** Mean amounts of money returned per round in Study 1.

	Baseline values		First observed values		Second observed values		Final values	
	*M*	*SD*	*M*	*SD*	*M*	*SD*	*M*	*SD*
CC (*N* = 25)	12.29	4.11	11.98	5.20	11.36	4.33	11.61	5.05
CS (*N* = 25)	11.44	4.10	11.32	4.02	9.10	4.80	10.30	4.69
SC (*N* = 25)	10.68	4.59	6.88	4.79	6.56	4.50	7.24	4.94
SS (*N* = 25)	11.59	4.27	7.86	5.29	6.26	5.35	6.28	5.24

We performed a 2 × 2 analysis of variance (ANOVA) on the final cooperativeness measure with two between–subject factors (cooperative first vs. selfish first; cooperative second vs. selfish second).We found that participants in the cooperative first condition cooperated more (*M* = 10.95, *SD* = 4.87) than those in the selfish first condition (*M* = 6.76, *SD* = 5.07), F(1,96) = 17.63, *p* < 0.001,$\eta_{p}^{2}$ = 0.155; participants in the cooperative second condition also cooperated more (*M* = 9.42, *SD* = 5.42) than those in the selfish second conditions (*M* = 8.29, *SD* = 5.33), but this difference was not significant, *F*(1,96) = 1.30, *p* > 0.250; the interaction was also not significant, *Fs* < 1, *p* > 0.250. This results show the primacy effect hypothesis is stronger than the recency effect, the environment participants exposed to first has a bigger effect on participants’ subsequent behaviors than the environment participants exposed to next. Moreover, we conducted an independent-samples *t*-test revealed that participants in the C-C condition behaved more cooperatively (*M* = 11.61, *SD* = 5.05) than did participants in the S-S condition (*M* = 6.28, *SD* = 5.25), *t*(48) = 3.66, *p* < 0.001. Observing cooperative behaviors leads to more cooperative behaviors and observing selfish behaviors leads to more selfish behaviors. Finally, participants in the C-S condition are more cooperative (*M* = 10.24, *SD* = 4.69) than those in the S-C condition (*M* = 7.29, *SD* = 4.94). *t*(48) = 2.24, *p* = 0.030. This result also lends support to the importance of the primacy effect.

## Study 2: Counteracting Primacy

We conducted two additional experiments to examine how the primacy effect can be counteracted by recency exposure. In study 2a, we tested how long an individual needs to be immersed in a cooperative environment in order for the more recent exposure to counteract the negative effect of exposure to a selfish environment first. In study 2b, we want to know the analog: when an individual is exposed to a cooperative environment first, how long an exposure to a subsequent selfish environment is needed in order to counteract the primacy effect.

### Method

#### Participants

In study 2a, 58 participants from Sun Yat-sen University participated in the experiment for monetary payoff. Participants received a ¥10 payment plus 1% of the payment earned from the economic game they played. One participant was excluded because of a computer malfunction during the game. Fifty seven participants (39 females, mean age = 22.79 years, *SD* = 3.60) completed this study. Participants were randomly assigned to one of two conditions: S-C-C-C (a selfish environment first and then three times cooperative environments) and S-N-N-N (a selfish then three times neutral environment). This study was approved by the Sun Yat-Sen University Human Research Ethics Committee. All participants were fully informed of the nature of the experiment and signed an informed consent to participate.

In study 2b, fifty (22 females, mean age = 22.10 years, *SD* = 2.74) participants were recruited from Sun Yat-sen University to complete this study. Participants received a ¥10 payment plus 1% of the payment earned from the economic game they played. Participants were also randomly assigned to one of two conditions: C-S-S-S (a cooperative environment and then three consecutive selfish environment), and the control condition C-N-N-N (a cooperative and then three subsequent neutral environment). This study was approved by the Sun Yat-Sen University Human Research Ethics Committee. All participants were fully informed of the nature of the experiment and signed an informed consent to participate.

We followed similar procedures as in Study 1. Participants played trust games as the Receiver with students from three different universities. They played with students from each university and each round was with a different person. Specifically, they played with students from University 1 for 10 rounds, with those from University 2 for 10 rounds, and then with those from University 3 for thirty rounds. For University 2 and University 3, we had extra information to show to participants before participants played with students from these two universities. We told participants we recorded how students from University 2 and University 3 play the game among themselves. So before playing with students from University 2, participants viewed ten rounds of previous game playing among students from University 2 (i.e., both Investor and Receiver were from University 2). The same was true with University 3: participants viewed 10 rounds of game playing among University 3 students before they themselves actually played ten rounds of games with those from University 3. Then they viewed another ten rounds of games for University 3 again and played ten rounds with University 3 again. Finally, they view 10 rounds of play for University 3 for the third time, and played with University 3 for the third time. Taken together, participants viewed 30 rounds from University 3 and played 30 rounds with students from University 3.

Participants were told specifically the students they played the game with were different from those viewed in the video, but they were from the same university.

Specifically, for participants in the C-S-S-S condition, Receivers from University 2 typically behave cooperatively and Receivers from University 3 typically behave selfishly in the video for participants to view. For those in the S-C-C-C condition, University 2 was selfish and University 3 was cooperative. For those in the S-N-N-N and C-N-N-N conditions, University 3 was designed to be a neutral environment.

Similar to study 1, participants played 10 rounds of games with University 1. They decided how much they were going to return ten times. Their uninformed decisions from University 1 were used for the basis to construct a cooperative environment, a selfish environment, and a neutral environment for them to view later. The cooperative and selfish environments were constructed in the same way. The neutral environment was constructed by selecting two decisions to add 1 yuan and selecting another two decisions to subtract 1 yuan. The rest of the decisions remained the same. The presentation order in the video view is randomized so no participants suspected the manipulation.

##### Dependent measure

Our dependent measures were now five cooperativeness indexes, measured by the average amount returned to students from each of the three universities across 10 rounds.

### Results

We performed an analysis-of-covariance (ANCOVA) using the average amount returned in the three University 3 trials as the dependent variables and the two conditions as between-subjects factors, and the average amount participants returned to investors from University 2 as a covariate.

In the study 2a, we compared participants who observed three cooperative environments in the experimental condition to participants in the control condition, who observed three neutral environments.

**FIGURE 4:**
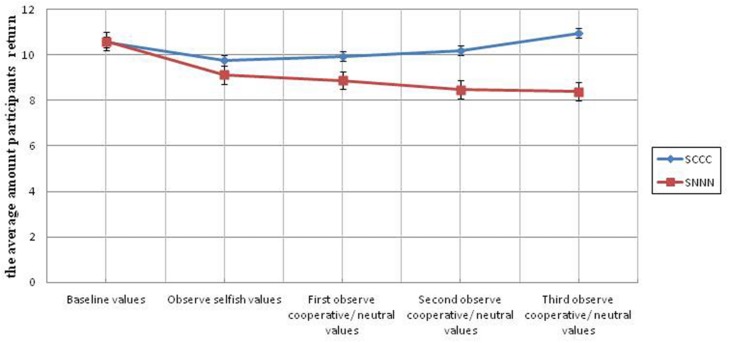
**Average return amounts of participants from each of the three universities across 10 rounds in Study 2a.** Error bars represent the standard error of the mean.

There is a significant increase in cooperativeness in the experimental condition (SCCC) compared to the control condition (SNNN) only in the third round of cooperative exposure, *F*(1,54) = 4.66, *p* = 0.035, $\eta_{p}^{2}$ = 0.079; there was no significant difference, in the first or the second cooperative treatment rounds, *F*(1,54) = 0.65, *p* > 0.250, and *F*(1,54) = 2.46, *p* = 0.12, respectively (see **Figure 4**, **Table 2**).

**Table 2:** Mean amounts of money returned per round in Study 2a.

	Baseline values		Observe selfish values		First observe cooperative/neutral values		Second observe cooperative/neutral values		Third observe cooperative/neutral values	
	*M*	*SD*	*M*	*SD*	*M*	*SD*	*M*	*SD*	*M*	*SD*
SCCC (*N* = 29)	10.57	4.85	9.57	5.22	9.94	5.79	10.21	6.06	10.96	6.59
SNNN (*N* = 28)	10.60	4.91	9.14	5.19	8.89	5.73	8.48	5.49	8.38	5.81

**FIGURE 5:**
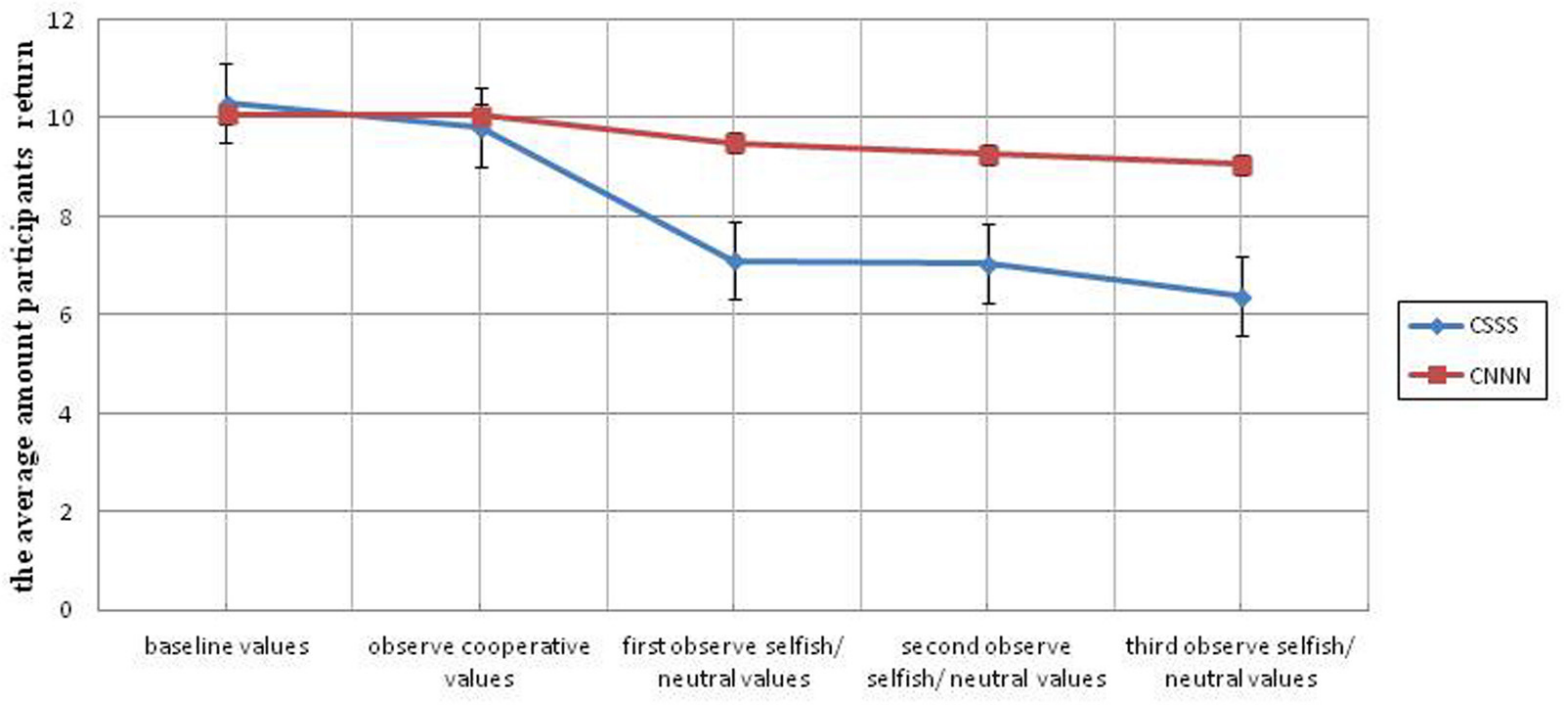
**Average return amounts of participants from each of the three universities across ten rounds in Study 2b.** Error bars represent the standard error of the mean.

In study 2b, there is a significant difference in the experimental condition (CSSS) compared to the control condition (CNNN) across all three selfish treatment environments. There is a significant difference after the first exposure to a selfish environment, *F*(1,47) = 6.95, *p* = 0.011, $\eta_{p}^{2}$ = 0.129; and this difference persists for the second and third treatment exposures as well, *F*(1,47) = 6.05, *p* = 0.0018, $\eta_{p}^{2}$ = 0.114 and *F*(1,47) = 6.69, *p* = 0.013, $\eta_{p}^{2}$ = 0.125, respectively (see **Figure 5**, **Table 3**).

**Table 3:** Mean amounts of money returned per round in Study 2b.

	Baseline values		Observe cooperative values		First observe selfish/neutral values		Second observe selfish/neutral values		Third observe selfish/neutral values	
	*M*	*SD*	*M*	*SD*	*M*	*SD*	*M*	*SD*	*M*	*SD*
CSSS (*N* = 25)	10.32	5.04	9.81	5.28	7.11	5.05	7.05	5.02	6.37	5.34
CNNN (*N* = 25)	10.08	4.62	10.07	5.30	9.50	4.56	9.26	5.02	9.06	4.74

### Discussion

In study 1, we test Hypothesis 1 and find confirmatory evidence for the primary effect and suggestive evidence for the recency effect. We examined how participants behave in a neutral final round after exposure to two rounds where the environment was manipulated to be either cooperative or selfish. Holding the second environment fixed, participants who saw a cooperative first environment were more likely to cooperate in the neutral final round. Holding the second environment fixed, participants who saw a selfish first environment were more likely to be selfish in the neutral final round. Taken together, this provides evidence for the primacy effect. As for the recency effect, holding the first environment fixed, participants were more likely to conform to the behavior they witnessed in the second round environment, but this was only statistically significant when the first round they observed was cooperative. When the primacy and recency effects were considered together, the recency effect was significantly smaller than the primacy effect, with no significant interaction between the two.

The fact that the recency effect was only observed in response to the cooperative exposure is notable because the immediate response to a selfish environment was much stronger than the immediate response to a cooperative environment. When subjects thought they were interacting against selfish partners, their rate of cooperation declined significantly from their baseline. However, when subjects thought they were interacting with more cooperative partners, their rate of cooperation stayed the same. This is not to say being in a cooperative environment had no impact. As we see in Experiment 2, as well as in prior literature (e.g., Isaac et al., 1994), left alone or in a neutral environment, people tend to behave more selfishly with repeated play, so the fact that they maintained the same level of cooperativeness shows some impact of exposure to cooperation. However, the immediate response to a cooperative environment is still much smaller than the immediate response to a selfish environment. It is remarkable, then, that licensing of selfish behavior is at least in part context specific. I may feel more justified in acting selfishly when playing against selfish partners, but when it comes to our measure of the recency effect, it is exposure to cooperation that is more likely to carry over to a new environment.

First exposure to the norms of an environment is important. In this setting, the novelty of the first exposure to our environmental manipulation was able to shift participants from the behavioral norms they brought into the lab with them, as evidenced by how post-treatment behavior diverges from pre-treatment behavior. This is notable because the effect of primacy lasts through intervening rounds of exposure to another environment. The point in time when someone is first placed in an unfamiliar situation and given an unfamiliar task is the most effective time to imprint new cultural norms. This first exposure has lasting impact but it could potentially be counteracted by the most recent environmental exposure. In study 1, the effect of the most recent environment was dwarfed by the effect of the first environment. Therefore, we turn to the results in study 2 to examine how long it takes for participants to assimilate to environmental norms they are being exposed to.

Study 2 has two parts. The first placed subjects in a cooperative environment first, and then counteracted that primacy effect by exposing them to three consecutive selfish environments. We compared their behavior with a control group who was also exposed to a cooperative environment but then subsequently exposed to three neutral environments. The second part of study 2 placed subjects in a selfish environment first, and then counteracted that selfishness by exposing them to three cooperative environments. We compared those results to a neutral control as well.

We found that the primacy effect can indeed be counteracted by recent exposure either through prolonged exposure or through selfish exposure. In the selfish first condition, three consecutive exposures to cooperative environments completely neutralized the primacy effect. By the last round the most recent environmental exposure came to dominate. The effect was even stronger in the cooperative condition. It only took one round of exposure to a selfish environment for the recency effect to come to dominate. The impact of exposure to a more selfish environment lasted longer and influenced behaviors to a greater extent than exposure to a more cooperative environment. One limitation of our study is that we can only compare the strength of our particular selfishness induction and our particular cooperativeness induction. What we can say is that because the cooperative and selfish environments were constructed symmetrically, we can make the general point that if a person observes some amount of more selfish behavior, versus an equal amount of more cooperative behavior, exposure to more selfish behavior is more potent. In our study, the selfish environment was more potent in the short run. It took only one exposure to a selfish environment to counteract the primacy effect of a cooperative environment by inspiring selfish play; one exposure to a cooperative environment led to almost no change in behavior. However, in the long run, after exposing subjects to three consecutive selfish or cooperative environments, cooperation and selfishness were similarly effective at reversing the primacy effect.

Two mechanisms are likely at work. There is the effect of the social norm itself, which through the conformity mechanisms discussed earlier; people shift their behaviors to match others’ behaviors that they observe (Cialdini et al., 2006). However, there is a second mechanism which produces an asymmetry between cooperative exposures versus selfish exposure. Moral regulation and moral licensing (Sachdeva et al., 2009) gives people permission to act selfishly if they believe they had performed a moral action in the past. Exposure to an environment where others acted more selfishly makes one feel good about their past behavior and therefore licenses the participant to act selfishly in future interactions. The theory of moral regulation applies in the other direction as well—learning one acted more selfishly than others makes people feel guilty and encourages more cooperative behavior. There is an asymmetry here, however. As observed in Ho et al. (2015) moral regulation is more likely to license selfish behavior than it is to engender cooperative behavior—we are more likely to use the information about others to feel good about ourselves than to feel guilty. While we did observe an increase in cooperation when exposed to cooperative environments, the effect was small relative to the increase of selfishness while playing in a selfish environment. However, as noted above, participants were more likely to carry the effects of cooperative norms into subsequent environments. The effects of cooperation accreted slowly over time, while the impact of selfish exposure tended to happen all at once.

Taken together, our findings provide guidance for establishing, maintaining, merging, or changing an organizational culture as new individuals or groups are integrated. Attention must be given to the initial environment new members are exposed to because the primacy effect is strong. Additionally, exposure to selfish environments must be carefully monitored, as the effect of selfish norms will be more difficult to counteract; in the short run, cooperative environments were largely ineffective. With repeated exposure, however, cooperative norms can take hold, suggesting the potential for long run organizational change.

## Author Contributions

XZ, YL, and BH designed the study. Data were collected and analyzed by YL. XZ, YL, and BH and drafted the manuscript. All authors approved the final version of the manuscript for submission.

## Conflict of Interest Statement

The authors declare that the research was conducted in the absence of any commercial or financial relationships that could be construed as a potential conflict of interest.
